# Conserved Gene Order and Expanded Inverted Repeats Characterize Plastid Genomes of Thalassiosirales

**DOI:** 10.1371/journal.pone.0107854

**Published:** 2014-09-18

**Authors:** Jamal S. M. Sabir, Mengjie Yu, Matt P. Ashworth, Nabih A. Baeshen, Mohammad N. Baeshen, Ahmed Bahieldin, Edward C. Theriot, Robert K. Jansen

**Affiliations:** 1 Department of Biological Sciences, Faculty of Science, King Abdulaziz University, Jeddah, Saudi Arabia; 2 Department of Integrative Biology, University of Texas at Austin, Austin, Texas, United States of America; Stazione Zoologica Anton Dohrn, Naples, Italy

## Abstract

Diatoms are mostly photosynthetic eukaryotes within the heterokont lineage. Variable plastid genome sizes and extensive genome rearrangements have been observed across the diatom phylogeny, but little is known about plastid genome evolution within order- or family-level clades. The Thalassiosirales is one of the more comprehensively studied orders in terms of both genetics and morphology. Seven complete diatom plastid genomes are reported here including four Thalassiosirales: *Thalassiosira weissflogii*, *Roundia cardiophora*, *Cyclotella* sp. WC03_2, *Cyclotella* sp. L04_2, and three additional non-Thalassiosirales species *Chaetoceros simplex*, *Cerataulina daemon*, and *Rhizosolenia imbricata*. The sizes of the seven genomes vary from 116,459 to 129,498 bp, and their genomes are compact and lack introns. The larger size of the plastid genomes of Thalassiosirales compared to other diatoms is due primarily to expansion of the inverted repeat. Gene content within Thalassiosirales is more conserved compared to other diatom lineages. Gene order within Thalassiosirales is highly conserved except for the extensive genome rearrangement in *Thalassiosira oceanica*. *Cyclotella nana*, *Thalassiosira weissflogii* and *Roundia cardiophora* share an identical gene order, which is inferred to be the ancestral order for the Thalassiosirales, differing from that of the other two *Cyclotella* species by a single inversion. The genes *ilvB* and *ilvH* are missing in all six diatom plastid genomes except for *Cerataulina daemon*, suggesting an independent gain of these genes in this species. The *acpP1* gene is missing in all Thalassiosirales, suggesting that its loss may be a synapomorphy for the order and this gene may have been functionally transferred to the nucleus. Three genes involved in photosynthesis, *psaE*, *psaI*, *psaM*, are missing in *Rhizosolenia imbricata,* which represents the first documented instance of the loss of photosynthetic genes in diatom plastid genomes.

## Introduction

Diatoms are unicellular organisms with delicate siliceous walls, forming a monophyletic group within the heterokont algae [1]–[4]. Most diatoms are photosynthetic and are responsible for one quarter of global net primary production, and they are the main biological mediators of the silica cycle in the oceans [5]. The completion of nuclear and plastid genome sequences for three diatoms, *Cyclotella nana* Hustedt [6] (formerly *Thalassiosira pseudonana* Hasle & Heimdal [7]), *Phaeodactylum tricornutum* Bohlin [8], and *Thalassiosira oceanica* Hasle [9], allowed the exploration of their evolutionary history in a genomic context. For example, one environmentally-driven gene transfer event has been reported in *T. oceanica*, where the *petF* gene encoding ferredoxin was transferred from the plastid to the nucleus [9]. Replacing the iron-sulfur protein ferredoxin by iron-free flavodoxin presumably contributed to the ecological success of *T. oceanica* in iron limited environments [9].

Understanding possible adaptive events such as the transfer of *petF* requires a dense taxon sampling of the trait of interest over a well-resolved phylogeny. The Thalassiosirales Glezer & Makarova are the only diatom order with a moderately well-resolved phylogeny that has been used to formally examine the evolution of ecological, morphological and genetic traits, particularly with regard to adaptation across marine and freshwater environments [10], [11].

Fifteen diatom plastid genomes have been sequenced so far [9], [12]–[17]. The overall organization of these genomes is conserved with all of them having a large single copy region (LSC), small single copy region (SSC), and two inverted repeats (IR). However, the plastid genomes range from ∼ 116 to 165 kb, and they show extensive genome rearrangements, gene loss, duplication and functional transfers of genes to the nucleus [16]. The first introns in diatom plastid genome were reported in the *rnl* and *atpB* genes of *Seminavis robusta* [15], and extrachromosomal plasmids were found in several diatom plastid genomes [15], [16].

In this study, plastid genome sequences are reported for four more thalassiosiralean diatoms (*Thalassiosira weissflogii* (Grunow) G. Fryxell & Hasle, *Cyclotella* (F.T. Kützing) A. de Brébisson sp. L04_2, *Cyclotella* (F.T. Kützing) A. de Brébisson sp. WC03_2 and *Roundia cardiophora* (Round) Makarova) and representatives of three other diatom orders, Chaetoceratales Round & Crawford (*Chaetoceros simplex* Ostenfeld), Hemiaulales Round & Crawford (*Cerataulina daemon* (Greville) Hasle in Hasle & Syvertsen) and Rhizosoleniales Silva (*Rhizosolenia imbricata* Brightwell). Gene content, genome size and gene order are compared across the genomes to better understand plastid genome evolution within Thalassiosirales.

## Materials and Methods

### Diatom strains and culture conditions

Seven diatom strains from different sources were examined (Table S1). There were no permissions required for those collection sites, and there are no endangered/protected diatoms. All DNA were extracted from cultured materials, several of which are already publicly available. *Cerataulina daemon*, *Roundia cardiophora* and *Rhizosolenia imbricata* were grown in marine f/2 medium [18] in a Percival model I-36LL incubation chamber (Percival, Boone, Iowa, USA) at 21°C; *Cyclotella sp. L04_2* and *Cyclotella sp. WC03_2* were grown in COMBO medium [19] on a window-lit lab bench; *Thalassiosira weissflogii* and *Chaetoceros simplex* were grown in f/2 medium [18] on a window-lit lab bench. The incubator was illuminated with fluorescent lights using a 12:12 hour light:dark photoperiod.

### DNA extraction

Diatom cells were pelleted in a Sorvall RC-5B refrigerated superspeed centrifuge (DuPont Company, Newton, CT, USA) for 20 minutes at 7649×g from a culture in the late logarithmic phase of growth. Cells were lysed using a PARR Cell Disruption Bomb (Parr Instrument Company, Moline, IL, USA) filled with nitrogen gas at 1500 psi. Isolation of DNA was performed following Doyle and Doyle [20] with modifications. Cetyl trimethylammonium bromide (CTAB) buffer was augmented with 3% PVP and 3% beta-mercaptoethanol (Sigma, St. Louis MO, USA). Organic phase separation was repeated until the aqueous fraction was clear. DNA pellets were resuspended in ∼200 µL DNase-free water. Following treatment with RNase A (ThermoScientific, Lafayette, CO, USA) samples were again subjected to phase separation with chloroform, and DNA was recovered by ethanol precipitation. Samples were resuspended in DNase-free water, evaluated for concentration by NanoDrop and stored at −20°C.

### DNA sequencing and genome assembly

Paired-end (PE) libraries with insert sizes of 400 bp were prepared at the Genome Sequence and Analysis Facility (GSAF) at the University of Texas at Austin. Illumina HiSeq 2000 paired-end platform (Illumina, San Diego, CA, USA) was used to sequence total genomic DNA. The PE Illumina reads were assembled with Velvet v.1.2.08 [21], [22] using multiple *k*-mers ranging from 71 to 83. Plastid contigs were identified by BLAST analyses of the assembled contigs against published diatom plastid genomes from NCBI. The boundaries between inverted repeats and single copy regions were confirmed bioinformatically or using PCR and Sanger sequencing. The latter two techniques were also utilized to fill gaps in the plastid genome sequences. The PCR primers used for Sanger sequencing were designed by Primer3 [23] in Geneious R6 v.6.1.6 [24] (Table S2).

### Genome annotations and analyses

Plastid genomes were annotated using Dual Organellar GenoMe Annotator (DOGMA) [25], followed by manual corrections for start codons using Geneious R6 v.6.1.6. tRNA genes were predicted using DOGMA [25] and tRNAscan-SE 1.21 [26]. Boundaries of rRNA genes, tmRNA *ssra* gene and signal recognition particle RNA *ffs* gene were delimited by direct comparison to sequenced diatom orthologues with Geneious R6 v.6.1.6 [24]. Circular plastid genome maps were generated with Organellar GenomeDraw (OGDraw) [27]. Repeated sequences were identified by performing BlastN v.2.2.28+ comparisons of each plastid genome against itself with an e-value cutoff of 1e^−10^ and at least 90 percent sequence identity. Annotated plastid genomes are available from GenBank using accession numbers KJ958479 – KJ958485. Genome rearrangements were estimated with MAUVE after eliminating one copy of the inverted repeat [28]. Numbers of genome inversions were inferred by GRIMM [29].

### Identification of genes transferred to the nucleus and signal peptides

Genes absent from plastid genomes were searched for by BLAST searches in *Cyclotella nana* nuclear genome against assembled contigs of transcriptome assemblies of *T. weissflogii* (MMETSP0878) and *Rhizosolenia setigera* (MMETSP0789) from the Marine Microbial Eukaryote Transcriptome Sequencing Project (MMETSP) website (http://marinemicroeukaryotes.org/) and nuclear assembly of *T. oceanica* (http://www.ncbi.nlm.nih.gov/Traces/wgs/?val=AGNL01#contigs) using BLASTN with an e-value cutoff of 1e^−10^. The previous reported nuclear copy of *acp* gene in *Cyclotella nana* (XM_002290970) was used as the query sequence to search for the missing *acp* genes. SignalP was used to predict signal peptides and cleavage sites [30].

### Phylogenetic analysis

Sequences of 20 plastid genes (*psaA*, *psbC*, *petD*, *petG*, *atpA*, *atpG*, *rbcL*, *rbcS*, *rpoA*, *rpoB*, *rps14*, *rpl33*,*rnl*, *rns*, *ycf89*, *sufB*, *sufC*, *dnaK*, *dnaB*, *clpC*) from 22 diatom taxa were aligned with MAFFT [31]. This included 15 published diatom plastid genomes and the seven genomes sequenced in this study. All sequences were included, and protein-coding genes were partitioned by gene and codon position. A maximum likelihood tree was constructed with RAxML7.2.8 [32], using the substitution model GTR+G+I and “-f a” option, and 1000 bootstrap replicates were performed to evaluate support for clades.

## Results

### 1. General features of plastid genomes

**Figure 1:**
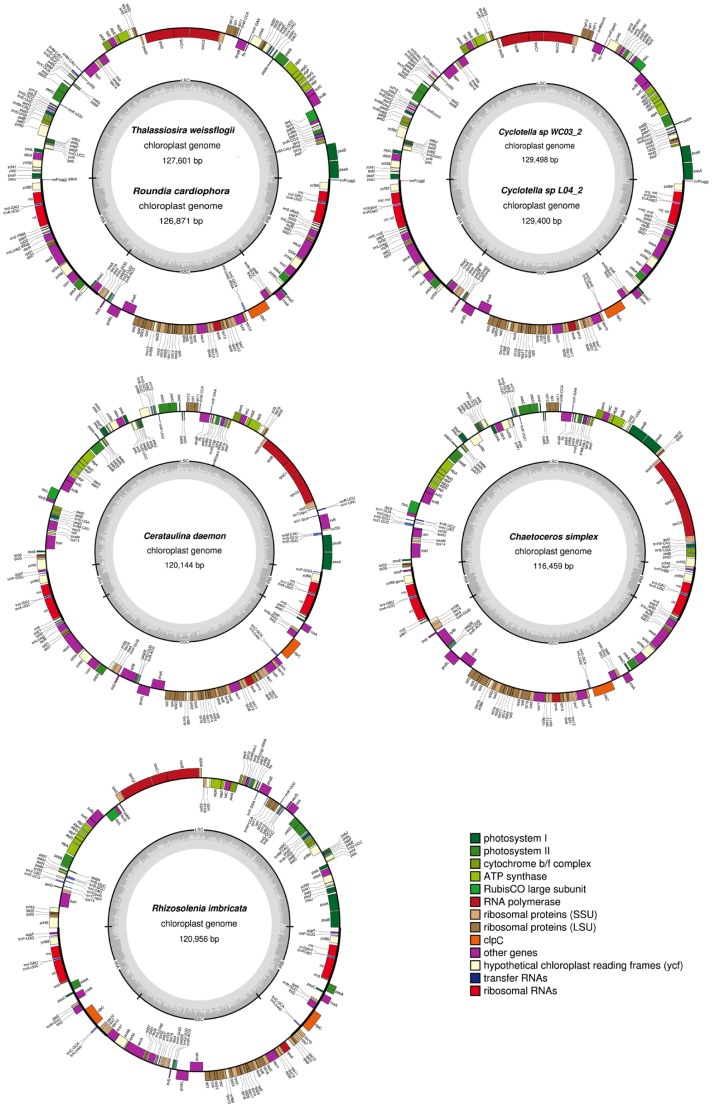
Plastid genome maps of seven newly sequenced diatom species. Species that share the same circular map have the same gene order. Genes on the outside are transcribed clockwise; those on the inside counterclockwise. The ring of bar graphs on the inner circle display GC content in dark grey.

All seven sequenced plastid genomes mapped as single circles with two IRs dividing the genome into LSC and SSC regions (Figure 1). The genomes are compact and lack introns. The three rRNA subunits (5S, 16S and 23S) are in the IR. Twenty-seven tRNAs together with two other RNAs, transfer-messenger RNA (*ssra*) and plastid signal recognition particle RNA (*ffs*), are found in all genomes. Nucleotide composition is highly conserved, with G+C content ranging from 30–32% (Table S3). Four pairs of overlapping genes are present in the seven diatom genomes; *sufC-sufB* by 1 bp; *psbD-psbC* by 53 bp; *atpD-atpF* by 4bp versus 1 bp in *Rh. imbricata*; and *rpl4-rpl23* by 17 bp in the two the *Cyclotellas* versus 8 bp in the other species (Table S3). The number of protein-coding genes ranges from 122 to 130. All protein-coding genes use the standard plastid-bacterial genetic code except for *psbC* in *Ro. cardiophora*, which uses ACG as the start codon instead of ATG. General features of the seven plastid genomes are compared with the two published thalassiosiralean genomes in Table S3.

### 2. Gene loss

**Figure 2:**
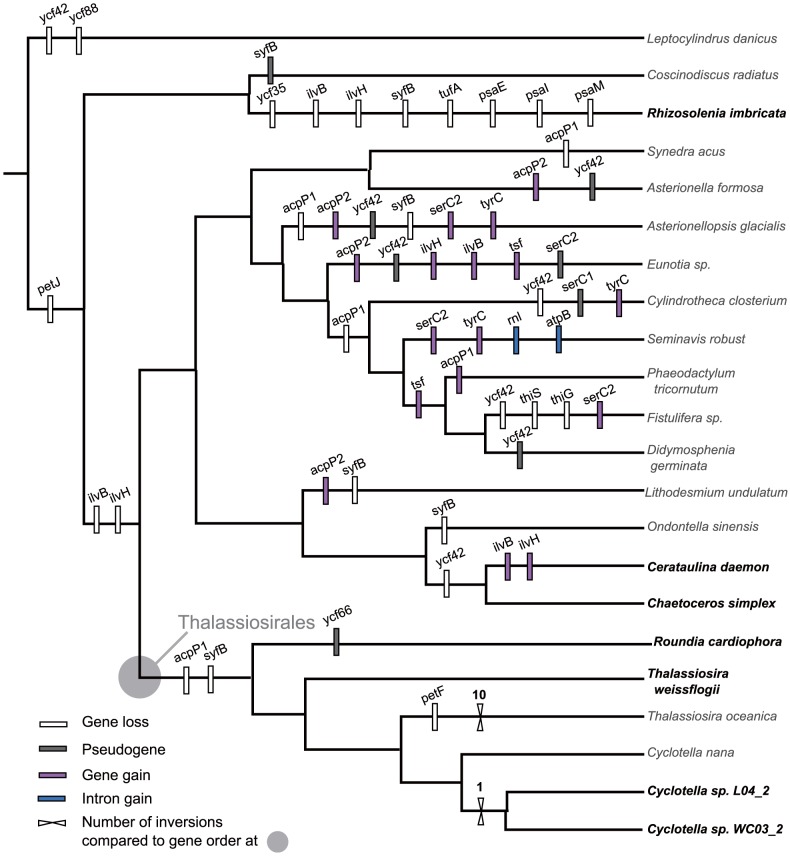
Phylogeny of Thalassiosirales and other diatom species based on twenty plastid protein-coding genes with gene/intron loss and plastid genome rearrangement events mapped on the branches. Number of genome inversions within Thalassiosirales were estimated based on Thalassiosirales ancestral genome using GRIMM [29]. Taxa in bold are new genomes sequenced in this study.

The protein-coding gene complement of the six Thalassiosirales plastid genomes is almost identical with 125 shared genes. A few notable exceptions were found. *ycf66* in *Ro. cardiophora* is a pseudogene as evidenced by several internal stop codons. The *acpP1* (acyl carrier protein) gene and the *syfB* (Phenylalanyl-tRNA synthetase) gene are missing in all Thalassiosirales (Figure 2; Table S4). *acpP1* is present in all three sequenced non-Thalassiosirales diatoms; however, *syfB* is missing only in the more distantly related *Rh. imbricata* (Figure 2; Table S4). The *ycf42* gene is missing in both *Ce. daemon* and *Ch. simplex*. The *ilvB* and *ilvH* genes, the large and small subunits of acetolactate synthase, are only found in *Ce. daemon* (Figure 2; Table S4). Several genes are missing from *Rh. imbricata*, including three photosynthetic genes (*psaE*, *psaI* and *psaM*), the protein translation elongation factor Tu (*tufA*), *syfB* and *ycf35*.

### 3. Functional gene transfer from plastid to nucleus

One ORF with 83.41% identity to the *Cyclotella nana* hypothetical plastid targeted acyl carrier protein gene *acp3* (XM_002290970) was found in the assembled transcriptome contig (MMETSP0878-20121228|7451_1) of *T. weissflogii*. The canonical signal peptide cleavage site ASAFVP, same as the signal peptide cleavage site of the *acp3* gene in *Cyclotella nana*, was found and indicated plastid targeting after cleaving between the endoplasmic reticulum (ER) signal peptide and transit peptide (Figure S1). However, SignalP did not indicate the presence of a signal peptide. BLAST analyses of the nuclear *acp3* gene of *Cyclotella nana* against the *T. oceanica* nuclear genome revealed one ORF with 86.64% identity. The canonical signal peptide cleavage site ASAFAP was found (Figure S1), and SignalP indicated peptide signaling to the ER. Searches for the missing *syfB* gene using gene sequences from the closely related species *Ce. daemon* and *Ch. simplex* against the nuclear genome of *Cy. nana* and *T. oceanica* and the transcriptome assembly of *T. weissflogii* did not identify any matches. Searching the annotated transcriptome data on the MMETSP website of a related species *Rhizosolenia setigera* Brightwell CCMP 1694 showed several contigs (MMETSP0789-20121207|1125_1, MMETSP0789-20121207|12246-1 *etc.*) annotated as elongation factor Tu domain or elongation factor Tu binding domain.

### 4. Genome size and repetitive DNA

**Figure 3:**
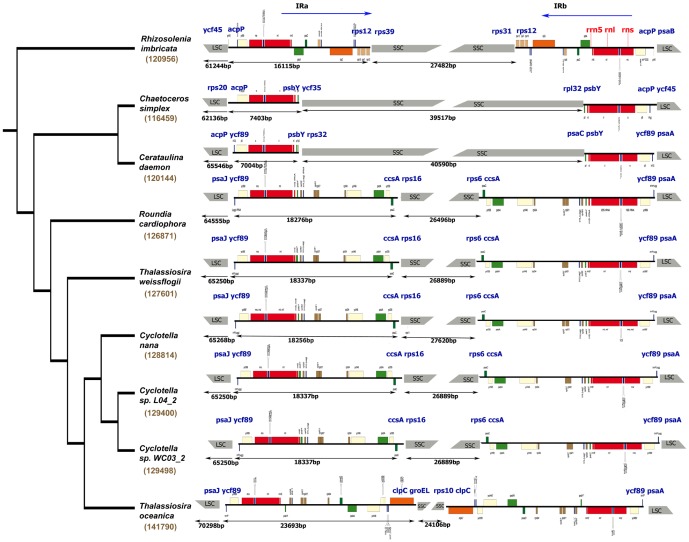
Comparison of inverted repeat boundaries in the seven diatom species newly sequenced for this study plus the two previously sequenced Thalassiosirales. Tree is that of Figure 2 with previously sequenced outgroup taxa pruned for visual simplicity. The numbers in brown indicate plastid genome size; the numbers in black below each genome fragment indicate the sizes of the LSC, IR and SSC, respectively. Protein coding genes at the IR boundaries are listed in blue. Three red gene blocks are *rrn5*, *rns* and *rnl*, respectively. Names in bold are Thalassiosirales. Underscored names are for taxa newly sequenced for this study.

The size of the seven sequenced diatom plastid genomes ranges from ∼ 116 kb in *Chaetoceros* to ∼ 129 kb in *Cyclotella* (Table S3). Plastid genomes of the Thalassiosirales are larger than the three non-Thalassiosirales species (*Ch. simplex*, *Ce. daemon* and *Rh. imbricata*, Table S3). The sizes of the LSC of the Thalassiosirales are similar to other diatoms sequenced here, however, the sizes of the SSC (24–27 kb) are smaller (27–40 kb) (Figure 3, Table S3). The IRs of Thalassiosirales tend to be larger, ranging from 18 to 23 kb, compared to 7 kb in *Ch. simplex* and *Ce. daemon* to 16 kb in *Rh. imbricata* (Figure 3, Table S3). The plastid genomes are compact with small intergenic spacer regions averaging 87–155 bp (Table S3). BLASTN analysis of each plastid genome against itself revealed only five short tandem repeats in Thalassiosirales with lengths ranging from 79 to 90 bp (Table S5).

The *rrnS-trnI-trnA-rnL-rrn5* gene cluster comprises the core of the IR. In Thalassiosirales, genes at the boundaries of IRs and single copy regions are the same, except for *T. oceanica*, which has an IR expanded through the *clpC* gene in SSC (Figure 3). The Chaetocerotales (*Ch. simplex*) and Hemiaulales (*Ce. daemon*) plastid genomes are smaller than the other diatoms examined. The IR of *Ch. simplex* is 7403 bp, which is slightly larger than the IR of *Ce. daemon* at 7004 bp (Figure 3). The IR of *Ch. simplex* includes one more gene (*acpP*) than *Ce. daemon*. The IR of Rhizosoleniales (*Rh. imbricata*) is larger than *Ch. simplex* and *Ce. daemon*.

### 5. Ancestral plastid genome organization of Thalassiosirales

**Figure 4:**
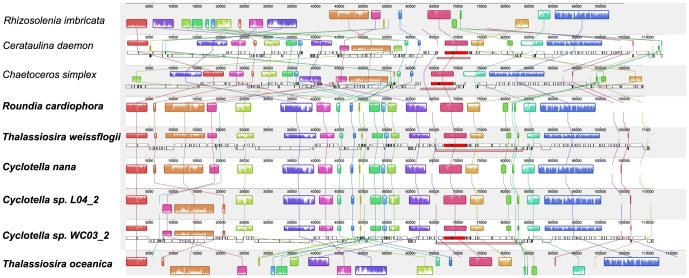
Gene order comparison of the plastid genomes of seven diatoms sequenced for this study plus previously sequenced Thalassiosirales. Alignments were performed in Geneious R6 [24]with mauveAligner [28]. Taxon names in bold are members of the Thalassiosirales. Names underscored are those sequenced for this study.

To reconstruct the ancestral plastid genome organization of Thalassiosirales, shared inversions and ancestral IR/SSC and IR/LSC boundaries were identified. The Mauve alignment identified thirty-two locally collinear blocks (LCBs) shared by the nine diatom plastid genomes examined (Table S6). Gene order within Thalassiosirales is very conserved, except for *T. oceanica* (Figure 4). *Cyclotella nana*, *T. weissflogii* and *Ro. cardiophora* have identical gene orders. Likewise, *Cyclotella* sp. L04_2 and *Cy.* sp. W03_2 have identical gene orders. The gene order of these two groups differs by only a single inversion of five adjacent LCBs (-19)(-15)(-14)(-9)(-10) between *rpl 19* and *rpl 20* in the LSC region (Table S6; Table S8; Figure 4). The plastid genome of *T. oceanica* is much more rearranged than other members of Thalassiosirales. GRIMM analysis estimated that ten inversions could explain the different gene orders between *Ro. cardiophora* and *T. oceanica* (Figure S2). Based on the most parsimonious reconstruction, the ancestral gene order of Thalassiosirales is the same as that of *Ro. cardiophora*, *T. weissflogii* and *Cy. nana*. The ancestral IR/LSC and IR/LSC boundaries in Thalassiosirales are shared by *Ro. cardiophora*, *T. weissflogii*, *Cy. nana*, *Cy.* sp. L04_2 and *Cy.* sp. WC03_2.

### 6. Genome rearrangements between Thalassiosirales and the other three diatoms sequenced

Twenty inversions were inferred between the ancestral Thalassiosirales condition and *Rh. imbricata* (Table S7). Fourteen inversions were inferred between the Thalassiosirales ancestral gene order and *Ce. daemon*, and seventeen inversions were inferred between the Thalassiosirales ancestral gene order and *Ch. simplex* (Table S7). Among those inversions two inverted gene blocks, (8) to (-8) and (23) to (-23), are shared by all three non-Thalassiosirales (Figure S3; Table S8). In addition, two inversions, (10)(9) to (-9)(-10) and (30)(31)(32)(27)(26)(25) to (-25)(-26)(-27)(-32)(-31)(-30), are shared by *Ce. daemon* and *Ch. simplex* (Figure S3; Table S8). *Chaetoceros simplex* and *Ce. daemon* gene orders are more similar to each other than either is to *Rh. imbricata* (Figure 4, Table S7). The most extensive genome rearrangement occurs between *T. oceanica* and *Rh. imbricata*, which differ by twenty-five inversions (Table S7).

## Discussion

The Thalassiosirales is a well-supported monophyletic diatom order common in marine, brackish, and freshwater habitats. Due to the monophyletic origin, we expect that the plastid genomes within this order will share many features in terms of gene content, genome size and gene order. All Thalassiosirales plastid genomes are very compact, lacking introns and having only a few short repeats. In contrast, genome organization of outgroup species varies considerably. The Thalassiosirales show a much higher level of conservation of genome organization compared to a recent comparison of a more phylogenetically diverse assemblage of diatoms [16]. Denser sampling of this order provides valuable insights into the dynamics of plastid genome evolution within a single order.

### Conserved gene content within Thalassiosirales

The plastid genomes of Thalassiosirales have 126–127 protein-coding genes, together with 3 rRNAs and 27 tRNAs (Table S3). Gene content variation is limited in the order with only few notable gene losses/transfers compared to other diatoms (Figure 2). The *acpP1* and *syfB* genes are absent from all Thalassiosirales. It is well known that plastid genes tend to undergo a sequential process of transfer from the plastid to the nucleus [33]. Centralized regulation of plastid metabolism in the nucleus has been suggested as a potential driving force for these transfers [9]. A nuclear encoded plastid targeted acyl carrier protein gene was reported in *Cyclotella nana* [14] and *Synedra acus* [13]. Previous research showed that a conserved amino acid motif AXAFXP at the cleavage site of the signal peptide was crucial for plastid targeting [34]. A nuclear encoded, plastid targeted acyl carrier gene was located in the nuclear genomes of *T. weissflogii* and *T. oceanica* with a canonical AXAFXP motif (Figure S1). Searching the transcriptome data of *Cyclotella meneghiniana* from the MMETSP website also revealed an ORF (CAMNT_0012963711) with 84.91% identity with the *acp3* gene in *Cyclotella nana,* and with an ASAFVP signal peptide cleavage motif indicating plastid targeting (data not shown). These results suggest that *acpP1* in Thalassiosirales likely represents a functional transfer from the plastid to the nucleus.

Transfer of *petF* from the plastid to the nucleus is unique to a single species of Thalassiosirales, *T. oceanica* [12]–[17]. It was suggested that this transfer may have been driven by an adaptation to a low iron environment [9]. To test whether this transfer is environmentally driven or limited to a single species, denser taxon sampling of species throughout the diatom phylogeny in different environments with varying amounts of iron is needed. The sequencing of the plastid genome of *Skeletonema*, the closest relative of *T. oceanica* [35], and other diatoms living in the open water with low iron concentration will enhance the understanding of the forces causing the transfer of the *petF* gene. Another possible gene loss/transfer within Thalassiosirales is *ycf66*, which is a pseudogene in *Ro. cardiophora* as suggested by the presence of several internal stop codons. However, more nuclear data are needed to test whether this gene is lost completely or it has been transferred to the nucleus.

### Variation of gene content in non-Thalassiosirales species

There are large differences in gene content in non-Thalassiosirales plastid genomes (Figure 2). The large and small subunits of acetolactate synthase, *ilvB* and *ilvH*, are reported present in all sequenced red algal plastid genomes [36]. There has been a history of repeated loss of these genes among the 16 diatom genomes [16]. Among the seven new plastid genomes reported here, *ilvB* and *ilvH* are absent in all species except *Cerataulina daemon*. The most parsimonious reconstruction of gene gain/losses suggests that these genes were reacquired independently by this species. More plastid genomes need to be sampled to better understand the loss/gain history of these genes across the diatom tree.

The *ycf42* gene is missing from the plastid genomes of both *Ce. daemon* and *Chaetoceros simplex*. This gene was reported lost from the plastid genome of *Fistulifera* sp. JPCC DA0580 [12], *Leptocylindrus danicus* and *Cylindrotheca closterium* [16], and has been pseudogenized in the plastid genomes of *Asterionellopsis glacialis*, *Asterionella formosa*, *Eunotia naegelii* and *Didymosphenia geminata* (Figure 2) [16]. More nuclear genome sequences are needed to determine whether *ycf42* has been transferred to the nucleus or has simply been lost.

The *ycf35* gene is missing from the *Rh. imbricata* plastid genome, representing the first case of the loss of this gene from a diatom. The *tufA* gene, encoding chloroplast protein synthesis elongation factor Tu, is also missing in *Rh. imbricata*. In the green algal ancestor of land plants, *tufA* was transferred from the plastid to the nucleus [37]. It is possible that *tufA* in *Rh. imbricata* has been functionally transferred to the nucleus but more nuclear data for this species is needed to confirm the transfer.

The most noteworthy gene losses are from the *Rh. imbricata* plastid genome where the three photosynthetic genes *psaE*, *psaI* and *psaM* are missing. It is well-known that parasitic prokaryotes and eukaryotes have experienced extensive genome size reduction due to loss of genes that are no longer functional [38], [39]. The plastid genome of non-photosynthetic euglenoid flagellate *Astasia longa* lost all photosynthetic genes from its plastid genome except for *rbcL* [40]. The non-photosynthetic parasitic flowering plant *Epifagus virginiana* only contains 42 genes, all genes for photosynthesis and chlororespiration, together with many tRNA and RNA polymerase genes have been lost [41]. But the loss of photosynthetic genes from plastid genomes of non-parasitic plants or algae is rare [42]. There are two possible explanations for the loss of *psaE*, *psaI* and *psaM* from the *Rh. imbricata* plastid genome. First, these genes may have been functionally transferred to the nucleus. Second, several studies have documented the presence of the endosymbiont, diazotrophic cyanobacterium *Richelia intracellularis* living within the siliceous frustules of several *Rhizosolenia* species, including *Rh. clevei* and *Rh. hebetat*a [43]–[45]. So, it is possible that the missing photosynthetic genes of *Rh. imbricata* have been horizontally transferred to the endosymbiont, similar to the situation that occurred in the sea slug [46]. However, without nuclear genome/transcriptome data for *Rh. imbricata* or evidence that a cyanobacterial endosymbiont genome has acquired these genes, it is not possible to determine which of these explanations is more likely.

### Genome size

Plastid genome size varies among diatoms, ranging from 116,251 bp in *Synedra acus* [13] to 165,809 bp in *Cylindrotheca closterium* [16]. Expansion/contraction/loss of the IR, gene loss and duplication, and reduced size of the introns and intergenic spacer regions are the major factors contributing to variation in genome size [33]. The large genome of *Cylindrotheca closterium* is mainly due to expanded intergenic spacer regions, which accounts for up to one quarter of the *Cylindrotheca* plastid genome [16]. It has been previously reported that the larger plastid genome size of *T. oceanica* compared to the *Cyclotella nana* is due to the expansion of the inverted repeat [9]. Thalassiosirales have larger plastid genomes than the three sequenced non-Thalassiosirales diatom in this study (Figure 1, Table S3), and most of the diatom species sequenced by Ruck *et al.* [16]. The low number of repeats and the larger IRs in Thalassiosirales compared other species (Table S3, Figure 3) indicates that their larger genome size is due to expansion of the IR.

### Genome rearrangements

Evolutionary events can alter the gene order through inversion, expansion/contraction of the IR, gene duplication/loss, and transposition. Inversions caused by recombination between repeated sequences are considered the major mechanism for gene order changes in plastid genomes [33]. There have been numerous rearrangements among published diatom genomes [16], however, only two species of Thalassiosirales were previously sampled. Completion of plastid genomes of four additional members of the Thalassiosirales and additional diatom species from other lineages shows that gene order within Thalassiosirales is highly conserved with the exception of *T. oceanica*. The sequenced Thalassiosirales plastid genomes have three different gene order patterns. The first and most common pattern is shared by *Ro. cardiophora*, *T. weissflogii* and *Cyclotella nana* and it represents the ancestral gene order for the order. The second pattern occurs in the two freshwater *Cyclotella* species, which have one inversion in the LSC region that may be a synapomorphy for this clade (Figure 2, Tables S6–S7). The third pattern is represented by *T. oceanica*, which is distinct from the rest of the Thalassiosirales. The genome has ten inversions relative to the ancestral genome arrangement for the order (Figure 2, Table S7). The IR boundary of *T. oceanica* is also distinct from the rest of the Thalassiosirales (Figure 3). IR boundary shifts are a common phenomenon [47] and is likely one of the factors contributing to the extensive rearrangements in *T. oceanica*. Alverson *et al.* [35] examined the molecular phylogeny of Thalassiosirales and found that *T. weissflogii* and *Cyclotella* species group together, while *T. oceanica* is more phylogenetically distant from the Thalassiosirales that share similar gene order. To examine whether the gene order change is gradual or punctuated, a wider sampling of plastid genomes across the rest of the Thalassiosirales will be needed to elucidate gene order evolution in this order.

## Supporting Information

Figure S1 **Processing sites of nuclear encoded plastid targeted acyl carrier protein.** The signal peptide (blue) is removed by signal peptidase (SPase) and the transit peptide (green) is removed by stromal processing peptidase (SPP). The signal peptide and transit peptide junction site show a canonical AXAFXP motif [48].
(PDF)

Figure S2 **Inversion events from the** ***Roundia cardiophora*** **plastid genome to** ***Thalassiosira oceanica*** **plastid genome.**
(PDF)

Figure S3 **Inversion events from the** ***Roundia cardiophora*** **plastid genome to three non-Thalassiosirales.**
(PDF)

Table S1 **Taxa used for plastid genome sequencing with source and GenBank accession numbers.**
(DOCX)

Table S2 **PCR Primers used for finishing diatom plastid genome sequencing and confirming boundaries between inverted repeats and single copy regions.**
(DOCX)

Table S3 **Plastid genome features of seven sequenced diatoms in comparison with** ***Cyclotella nana*** **and** ***Thalassiosira oceanica*** **.**
(DOCX)

Table S4 **Gene content comparison of seven sequence diatom plastid genomes with other published diatom plastid genomes.**
(XLSX)

Table S5 **Predicted repeat pairs in seven sequenced diatom plastid genomes.**
(DOCX)

Table S6 **The permutation of number coded Locally Colinear Block (LCB) for each plastid genome.** Negative number indicates an inversion of the given LCB.
(DOCX)

Table S7 **Pairwise number of inversions inferred by GRIMM.**
(DOCX)

Table S8 **Genes at the boundary of each Locally Colinear Block (LCB).**
(DOCX)
