# Supplementary material for: Conserved Gene Order and Expanded Inverted Repeats Characterize Plastid Genomes of Thalassiosirales
Source: PLoS One. 2014 Sep 18;9(9):e107854. doi: 10.1371/journal.pone.0107854 (PMC4169464; doi:10.1371/journal.pone.0107854)
Supplement: Figure S1 — Processing sites of nuclear encoded plastid targeted acyl carrier protein. The signal peptide (blue) is removed by signal peptidase (SPase) and the transit peptide (green) is removed by stromal processing peptidase (SPP). The signal peptide and transit peptide junction site show a canonical AXAFXP motif [48]. (PDF) [file pone.0107854.s001.pdf]

Supplementary figure 1. Processing sites of nuclear encoded plastid targeted acyl carrier protein.

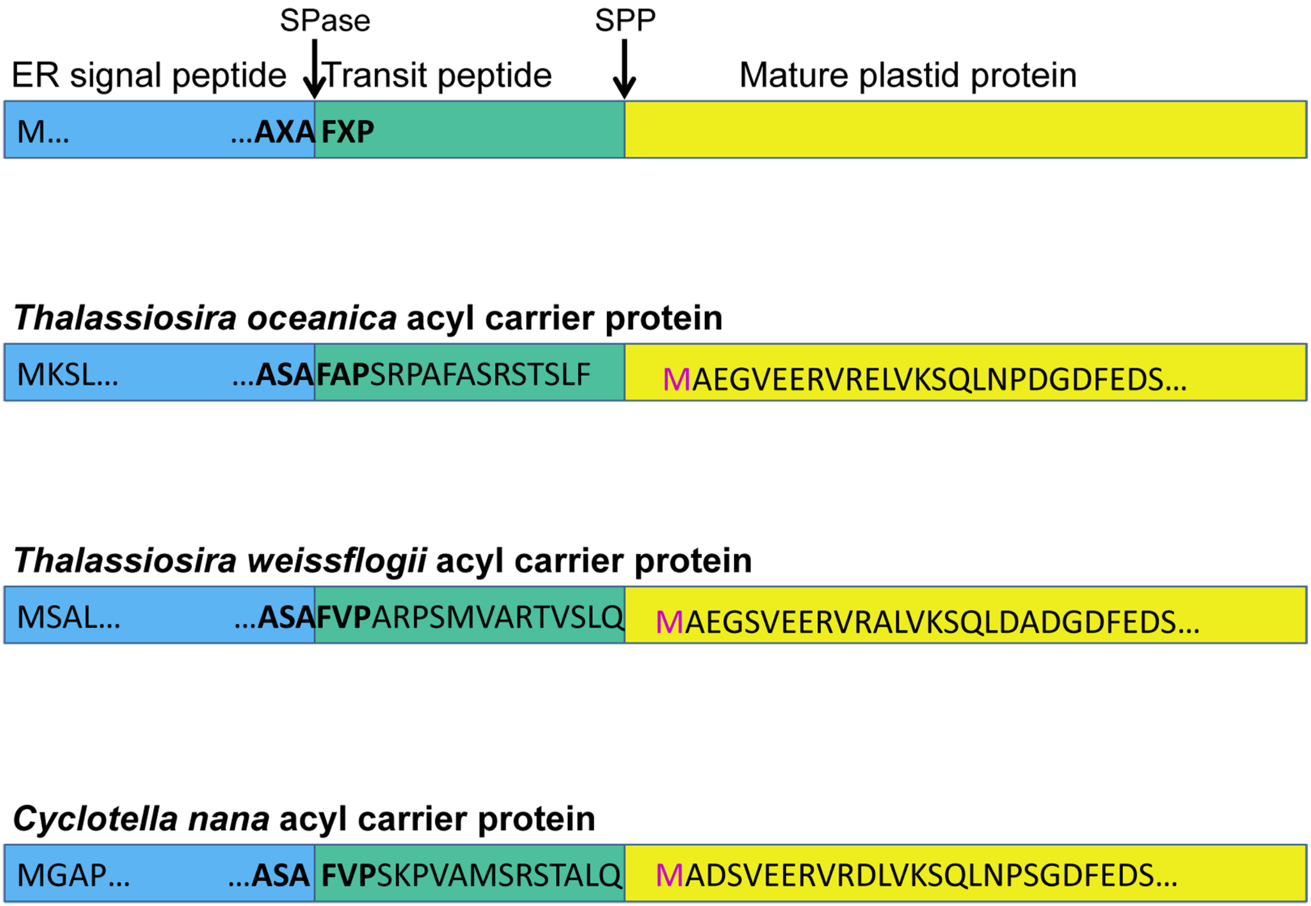

The signal peptide is indicaed in blue, the transit peptide is indicated in green.
SPase: signal peptidase.
SPP: Stromal processing peptidase
