# Supplementary material for: Conserved Gene Order and Expanded Inverted Repeats Characterize Plastid Genomes of Thalassiosirales
Source: PLoS One. 2014 Sep 18;9(9):e107854. doi: 10.1371/journal.pone.0107854 (PMC4169464; doi:10.1371/journal.pone.0107854)
Supplement: Figure S2 — Inversion events from the Roundia cardiophora plastid genome to Thalassiosira oceanica plastid genome. (PDF) [file pone.0107854.s002.pdf]

Supplementary figure 2. Inversion events from the *Roundia cardiophora* plastid genome to *Thalassiosira oceanica* plastid genome.

Step	Description	
0	*Roundia cardiophora*	1 10 9 14 15 19 20 8 12 11 6 18 17 16 13 5 7 4 3 2 21 29 28 22 23 24 30 31 32 27 26 25
1	Reversal	1 10 -14 -9 15 19 20 8 12 11 6 18 17 16 13 5 7 4 3 2 21 29 28 22 23 24 30 31 32 27 26 25
2	Reversal	1 10 -14 -9 15 19 20 8 12 11 -18 -6 17 16 13 5 7 4 3 2 21 29 28 22 23 24 30 31 32 27 26 25
3	Reversal	1 10 -14 -9 15 19 20 8 12 11 -18 -6 17 16 13 -7 -5 4 3 2 21 29 28 22 23 24 30 31 32 27 26 25
4	Reversal	1 10 -14 -20 -19 -15 9 8 12 11 -18 -6 17 16 13 -7 -5 4 3 2 21 29 28 22 23 24 30 31 32 27 26 25
5	Reversal	1 10 -14 -20 -19 -15 9 8 12 11 -18 -6 17 16 13 -7 -5 4 3 2 21 29 -24 -23 -22 -28 30 31 32 27 26 25
6	Reversal	1 10 -14 -20 -19 -15 9 8 12 11 -18 -6 17 16 13 -7 -5 4 3 2 21 29 -24 -23 -22 -28 30 -26 -27 -32 -31 25
7	Reversal	1 10 -14 -20 -19 -15 9 8 12 11 -18 -6 17 16 13 -7 -5 4 3 2 21 29 -24 -23 -22 -28 26 -30 -27 -32 -31 25
8	Reversal	1 10 -14 -20 -19 -15 9 8 12 11 -18 -6 17 16 13 -7 -5 4 3 2 21 29 -24 -23 -22 -28 26 -30 -25 31 32 27
9	Reversal	1 10 -14 -20 -19 -15 9 8 12 11 -3 -4 5 7 -13 -16 -17 6 18 2 21 29 -24 -23 -22 -28 26 -30 -25 31 32 27
10	Reversal	1 10 -14 -20 -19 -15 9 17 16 13 -7 -5 4 3 -11 -12 -8 6 18 2 21 29 -24 -23 -22 -28 26 -30 -25 31 32 27
	Thalassiosira oceanica	

Note: Only one IR is included in this analysis
