# Supplementary material for: Conserved Gene Order and Expanded Inverted Repeats Characterize Plastid Genomes of Thalassiosirales
Source: PLoS One. 2014 Sep 18;9(9):e107854. doi: 10.1371/journal.pone.0107854 (PMC4169464; doi:10.1371/journal.pone.0107854)
Supplement: Figure S3 — Inversion events from the Roundia cardiophora plastid genome to three non-Thalassiosirales. (PDF) [file pone.0107854.s003.pdf]

Supplementary figure 3. Inversion events from the *Roundia cardiophora* plastid genome to three non-Thalassiosirales.

Genome	Step	Description	Gene order
Roundia cardiophora	0	(Source)	1 10 9 14 15 19 20 8 12 11 6 18 17 16 13 5 7 4 3 2 21 29 28 22 23 24 30 31 32 27 26 25
	1	Reversal	1 10 9 14 15 19 20 -8 12 11 6 18 17 16 13 5 7 4 3 2 21 29 28 22 23 24 30 31 32 27 26 25
	2	Reversal	1 10 9 14 15 19 20 -8 12 11 6 18 17 16 13 5 7 4 3 2 21 29 28 22 -23 24 30 31 32 27 26 25
	3	Reversal	1 -9 -10 14 15 19 20 -8 12 11 6 18 17 16 13 5 7 4 3 2 21 29 28 22 -23 24 30 31 32 27 26 25
	4	Reversal	1 -9 -10 14 15 8 -20 -19 12 11 6 18 17 16 13 5 7 4 3 2 21 29 28 22 -23 24 30 31 32 27 26 25
	5	Reversal	1 -15 -14 10 9 8 -20 -19 12 11 6 18 17 16 13 5 7 4 3 2 21 29 28 22 -23 24 30 31 32 27 26 25
	6	Reversal	1 -15 -14 10 9 8 -20 -19 -18 -6 -11 -12 17 16 13 5 7 4 3 2 21 29 28 22 -23 24 30 31 32 27 26 25
	7	Reversal	1 -15 -14 10 9 8 -20 -19 -18 -6 -11 -12 17 16 -2 -3 -4 -7 -5 -13 21 29 28 22 -23 24 30 31 32 27 26 25
	8	Reversal	1 -15 -14 10 9 8 -20 -19 -18 -6 3 2 -16 -17 12 11 -4 -7 -5 -13 21 29 28 22 -23 24 30 31 32 27 26 25
	9	Reversal	1 -15 -14 10 9 8 -20 -19 -18 -6 3 2 -16 -17 12 11 -4 -7 -5 -13 21 29 28 22 -23 24 -25 -26 -27 -32 -31 -30
	10	Reversal	1 -15 -14 10 9 8 -20 -19 -18 -6 4 -11 -12 17 16 -2 -3 -7 -5 -13 21 29 28 22 -23 24 -25 -26 -27 -32 -31 -30
	11	Reversal	1 -15 -14 -4 6 18 19 20 -8 -9 -10 -11 -12 17 16 -2 -3 -7 -5 -13 21 29 28 22 -23 24 -25 -26 -27 -32 -31 -30
	12	Reversal	1 -15 -14 -4 6 18 19 20 -8 -9 -10 -11 -12 17 16 -2 -3 -7 -5 -13 21 29 28 22 30 31 32 27 26 25 -24 23
	13	Reversal	1 -17 12 11 10 9 8 -20 -19 -18 -6 4 14 15 16 -2 -3 -7 -5 -13 21 29 28 22 30 31 32 27 26 25 -24 23
Cerataulina daemon	14	Reversal (Destination)	1 -17 12 11 10 9 8 13 5 7 3 2 -16 -15 -14 -4 6 18 19 20 21 29 28 22 30 31 32 27 26 25 -24 23

Genome	Step	Description	Gene order
Roundia cardiophora	0	(Source)	1 10 9 14 15 19 20 8 12 11 6 18 17 16 13 5 7 4 3 2 21 29 28 22 23 24 30 31 32 27 26 25
	1	Reversal	1 10 9 14 15 19 20 -8 12 11 6 18 17 16 13 5 7 4 3 2 21 29 28 22 23 24 30 31 32 27 26 25
	2	Reversal	1 10 9 14 15 19 20 -8 12 11 6 18 17 16 13 5 7 4 -3 2 21 29 28 22 23 24 30 31 32 27 26 25
	3	Reversal	1 10 9 14 15 19 20 -8 12 11 6 18 17 16 13 5 7 4 -3 2 21 29 28 22 -23 24 30 31 32 27 26 25
	4	Reversal	1 -9 -10 14 15 19 20 -8 12 11 6 18 17 16 13 5 7 4 -3 2 21 29 28 22 -23 24 30 31 32 27 26 25
	5	Reversal	1 -9 -10 -15 -14 19 20 -8 12 11 6 18 17 16 13 5 7 4 -3 2 21 29 28 22 -23 24 30 31 32 27 26 25
	6	Reversal	1 -9 -10 -15 -14 19 20 -8 12 11 6 18 17 16 -2 3 -4 -7 -5 -13 21 29 28 22 -23 24 30 31 32 27 26 25
	7	Reversal	1 -9 -10 -15 -14 19 20 -8 12 11 6 18 17 16 -2 3 -4 -7 -5 -13 21 29 28 22 -23 24 -25 -26 -27 -32 -31 -30
	8	Reversal	1 -9 -10 -11 -12 8 -20 -19 14 15 6 18 17 16 -2 3 -4 -7 -5 -13 21 29 28 22 -23 24 -25 -26 -27 -32 -31 -30
	9	Reversal	1 11 10 9 -12 8 -20 -19 14 15 6 18 17 16 -2 3 -4 -7 -5 -13 21 29 28 22 -23 24 -25 -26 -27 -32 -31 -30
	10	Reversal	-9 -10 -11 -1 -12 8 -20 -19 14 15 6 18 17 16 -2 3 -4 -7 -5 -13 21 29 28 22 -23 24 -25 -26 -27 -32 -31 -30
	11	Reversal	-9 -10 -11 -1 -12 8 -20 -19 -18 -6 -15 -14 17 16 -2 3 -4 -7 -5 -13 21 29 28 22 -23 24 -25 -26 -27 -32 -31 -30
	12	Reversal	-9 -10 -11 -1 -12 8 -20 -19 -18 -6 -17 14 15 16 -2 3 -4 -7 -5 -13 21 29 28 22 -23 24 -25 -26 -27 -32 -31 -30
	13	Reversal	12 1 11 10 9 8 -20 -19 -18 -6 -17 14 15 16 -2 3 -4 -7 -5 -13 21 29 28 22 -23 24 -25 -26 -27 -32 -31 -30
	14	Reversal	12 1 11 10 9 8 -20 -19 -18 -6 -17 14 15 16 -2 3 -4 -7 -5 -13 21 30 31 32 27 26 25 -24 23 -22 -28 -29
	15	Reversal	12 1 11 10 9 8 13 5 7 4 -3 2 -16 -15 -14 17 6 18 19 20 21 30 31 32 27 26 25 -24 23 -22 -28 -29
	16	Reversal	-7 -5 -13 -8 -9 -10 -11 -1 -12 4 -3 2 -16 -15 -14 17 6 18 19 20 21 30 31 32 27 26 25 -24 23 -22 -28 -29
Chaetoceros simplex	17	Reversal (Destination)	-4 12 1 11 10 9 8 13 5 7 -3 2 -16 -15 -14 17 6 18 19 20 21 30 31 32 27 26 25 -24 23 -22 -28 -29

Genome	Step	Description	Gene order
Roundia cardiophora	0	(Source)	1 10 9 14 15 19 20 8 12 11 6 18 17 16 13 5 7 4 3 2 21 29 28 22 23 24 30 31 32 27 26 25
	1	Reversal	-1 10 9 14 15 19 20 8 12 11 6 18 17 16 13 5 7 4 3 2 21 29 28 22 23 24 30 31 32 27 26 25
	2	Reversal	-1 10 9 14 15 19 20 -8 12 11 6 18 17 16 13 5 7 4 3 2 21 29 28 22 23 24 30 31 32 27 26 25
	3	Reversal	-1 10 9 14 15 19 20 -8 12 11 6 -18 17 16 13 5 7 4 3 2 21 29 28 22 23 24 30 31 32 27 26 25
	4	Reversal	-1 10 9 14 15 19 20 -8 12 11 6 -18 17 -16 13 5 7 4 3 2 21 29 28 22 23 24 30 31 32 27 26 25
	5	Reversal	-1 10 9 14 15 19 20 -8 12 11 6 -18 17 -16 13 5 7 4 3 2 21 29 28 -22 23 24 30 31 32 27 26 25
	6	Reversal	-1 10 9 14 15 19 20 -8 12 11 6 -18 17 -16 13 5 7 4 3 2 21 29 28 -22 -23 24 30 31 32 27 26 25
	7	Reversal	-1 10 9 -15 -14 19 20 -8 12 11 6 -18 17 -16 13 5 7 4 3 2 21 29 28 -22 -23 24 30 31 32 27 26 25
	8	Reversal	-1 10 9 -15 -14 19 20 -11 -12 8 6 -18 17 -16 13 5 7 4 3 2 21 29 28 -22 -23 24 30 31 32 27 26 25
	9	Reversal	-1 10 9 -15 -14 19 20 -11 -12 8 6 -18 17 -16 13 5 7 4 3 2 21 29 28 -24 23 22 30 31 32 27 26 25
	10	Reversal	-1 10 9 -15 -14 19 20 -11 -12 8 6 -18 17 -16 13 5 7 4 3 2 21 -22 -23 24 -28 -29 30 31 32 27 26 25
	11	Reversal	-1 10 9 -15 -14 19 20 -11 -12 8 6 -18 17 -16 13 5 7 4 3 2 21 -22 -23 24 -32 -31 -30 29 28 27 26 25
	12	Reversal	-1 -20 -19 14 15 -9 -10 -11 -12 8 6 -18 17 -16 13 5 7 4 3 2 21 -22 -23 24 -32 -31 -30 29 28 27 26 25
	13	Reversal	-1 -20 -19 14 15 12 11 10 9 8 6 -18 17 -16 13 5 7 4 3 2 21 -22 -23 24 -32 -31 -30 29 28 27 26 25
	14	Reversal	-1 -20 -19 14 15 12 11 10 9 8 6 -18 17 -16 13 5 7 4 3 2 21 -22 -23 24 -25 -26 -27 -28 -29 30 31 32
	15	Reversal	-1 -20 -19 14 15 16 -17 18 -6 -8 -9 -10 -11 -12 13 5 7 4 3 2 21 -22 -23 24 -25 -26 -27 -28 -29 30 31 32
	16	Reversal	-1 -20 -19 -18 17 -16 -15 -14 -6 -8 -9 -10 -11 -12 13 5 7 4 3 2 21 -22 -23 24 -25 -26 -27 -28 -29 30 31 32
	17	Reversal	-1 -20 -19 -18 17 -16 -15 -14 -6 -5 -13 12 11 10 9 8 7 4 3 2 21 -22 -23 24 -25 -26 -27 -28 -29 30 31 32
	18	Reversal	-1 -20 -19 -18 17 -16 -15 -14 -6 -7 -8 -9 -10 -11 -12 13 5 4 3 2 21 -22 -23 24 -25 -26 -27 -28 -29 30 31 32
	19	Reversal	-1 -20 -19 -18 17 -16 -15 -14 -13 12 11 10 9 8 7 6 5 4 3 2 21 -22 -23 24 -25 -26 -27 -28 -29 30 31 32
Rhizosolenia imbricata	20	Reversal (Destination)	-1 -2 -3 -4 -5 -6 -7 -8 -9 -10 -11 -12 13 14 15 16 -17 18 19 20 21 -22 -23 24 -25 -26 -27 -28 -29 30 31 32

Blue box: Inversion shared by three non-Thalassiosirales (*Ce.daemon*, *Ch.simplex* and *Rh.imbricata*)

Red box: Inversion shared by *Ce.daemon* and *Ch.simplex*
