# Supplementary material for: Conserved Gene Order and Expanded Inverted Repeats Characterize Plastid Genomes of Thalassiosirales
Source: PLoS One. 2014 Sep 18;9(9):e107854. doi: 10.1371/journal.pone.0107854 (PMC4169464; doi:10.1371/journal.pone.0107854)
Supplement: Table S1 — Taxa used for plastid genome sequencing with source and GenBank accession numbers. (DOCX) [file pone.0107854.s004.docx]

**Supplementary table 1.** Taxa used for plastid genome sequencing with source and GenBank accession numbers.

| Taxon | Source/locality | GenBank Accession |
| --- | --- | --- |
| *Cerataulina daemon* | Atlantic coast, FL, USA  Approx. 26.9^o^ N, -80.0^o^ W | KJ958484 |
| *Chaetoceros simplex* | CCMP 200 | KJ958479 |
| *Cyclotella sp. L04_2* | Lake Ohrid, Macedonia | KJ958480 |
| *Cyclotella sp. WC03_2* | Waller Creek, TX, USA  30.12 ^o^ N, 97.43 ^o^ W | KJ958481 |
| *Thalassiosira weissflogii* | CCMP 1336 | KJ958485 |
| *Rhizosolenia imbricata* | Harbor Branch Oceanographic Institute boat dock, FL, USA  Approx. 27.5^o^ N, -80.3^o^ W | KJ958482 |
| *Roundia cardiophora* | Achang Reef, Guam, USA  13.249^o^ N, 144.697^o^ W | KJ958483 |

Abbreviation: CCMP (National Center for Culture of Marine Phytoplankton)
