# Supplementary material for: Conserved Gene Order and Expanded Inverted Repeats Characterize Plastid Genomes of Thalassiosirales
Source: PLoS One. 2014 Sep 18;9(9):e107854. doi: 10.1371/journal.pone.0107854 (PMC4169464; doi:10.1371/journal.pone.0107854)
Supplement: Table S2 — PCR Primers used for finishing diatom plastid genome sequencing and confirming boundaries between inverted repeats and single copy regions. (DOCX) [file pone.0107854.s005.docx]

**Table S2.** PCR Primers used for finishing diatom plastid genome sequencing and confirming boundaries between inverted repeats and single copy regions.

| Primer name | Sequence (5' → 3') |
| --- | --- |
| *Cerataulina_psaA_trnK_F* | TGA CCT GGT TGT GCC CAT TT |
| *Cerataulina_psaA_trnK_R* | ACC AAA CTG AGC TAT ATC CCG T |
| *Cerataulina_trnP_ycf45_f* | GAA CCT ACG ACA CCC TGG TC |
| *Cerataulina_trnP_ycf45_R* | ACA AGA GAT ATT AAA AAG GCA ACG A |
| *Cerataulina_psaC-psbX_F* | ACG AGT TGT TTC TGC GCC TA |
| *Cerataulina_psaC-psbX_R* | TGC ACC TGT TTT AAT CGC AGC |
| *Cerataulina_psbY_rbcR_F* | TGC ACC TGT TTT AAT CGC AGC |
| *Cerataulina_psbY_rbcR_R* | TCA GCA GCA CGT GTA AAG CT |
| *Cyclotella_L04_2_petG_F* | TCA AAT TGA TTT CCA CGA CGA T |
| *Cyclotella_L04_2_psaI_R* | ACC AAC AAG TGG TAC AAG AA |
