# Supplementary material for: Conserved Gene Order and Expanded Inverted Repeats Characterize Plastid Genomes of Thalassiosirales
Source: PLoS One. 2014 Sep 18;9(9):e107854. doi: 10.1371/journal.pone.0107854 (PMC4169464; doi:10.1371/journal.pone.0107854)
Supplement: Table S3 — Plastid genome features of seven sequenced diatoms in comparison with Cyclotella nana and Thalassiosira oceanica . (DOCX) [file pone.0107854.s006.docx]

**Table S3.** Plastid genome features of seven sequenced diatoms in comparison with *Cyclotella nana* and *Thalassiosira oceanica.*

|  | *T.*  *weissflogii* | *Cy. sp. L04_2* | *Cy. sp. WC03_2* | *Cy.*  *nana* | *T. oceanica* | *Ro. cardiophora* | *Ch. simplex* | *Ce. daemon* | *Rh. imbricata* |
| --- | --- | --- | --- | --- | --- | --- | --- | --- | --- |
| Size (bp) | **127,601** | **129,400** | **129,498** | **128,814** | **141,790** | **126,871** | **116,459** | **120,144** | **120,956** |
| SSC | 26,496 | 27,620 | 27,602 | 26,889 | 24,106 | 26,274 | 39,517 | 40,590 | 27,482 |
| LSC | 64,555 | 65,268 | 65,210 | 65,250 | 70,298 | 64,387 | 62,136 | 65,546 | 61,244 |
| IR | 18,276 | 18,256 | 18,261 | 18337 | 23,693 | 18,105 | 7,403 | 7,004 | 16,115 |
| G+C content | 30.8% | 30.3% | 30.0% | 30.7% | 30.4% | 31.0% | 32.1% | 31.2% | 31.8% |
| Protein coding genes | 127 | 127 | 127 | 127 | 126^a^ | 126^b^ | 128^c^ | 130^d^ | 122^e^ |
| rRNA genes | 3 | 3 | 3 | 3 | 3 | 3 | 3 | 3 | 3 |
| tRNA genes | 27 | 27 | 27 | 27 | 27 | 27 | 27 | 27 | 27 |
| Other RNAs | 2 | 2 | 2 | 2 | 2+flrn | 2 | 2 | 2 | 2 |
| genome coding for genes % | 85.18% | 85.25% | 84.88% | 85.56% | 79.67% | 85.16% | 87.34% | 84.56% | 79.46% |
| Gene density  (genes/kb) | 1.41 | 1.39 | 1.39 | 1.38 | 1.30 | 1.42 | 1.45 | 1.41 | 1.41 |
| Average IGS (bp) | 106.08 | 106.06 | 108.76 | 103.31 | 155.82 | 104.57 | 87.27 | 109.79 | 145.30 |
| Overlapping  genes | *sufC-sufB*: 1nt  *atpF-atpD:* 4nt  *psbC-psbD:* 53nt  *rpl4-rpl23:* 8nt | *sufC-sufB:* 1nt  *atpF-atpD:* 4nt  *psbC-psbD:* 53nt  r*pl4-rpl23:* 17nt | *sufC-sufB:*  1nt  *atpF-atpD:*  4nt  *psbC-psbD:* 53nt  *rpl4-rpl23:* 17nt | *sufC-sufB*: 1nt  *atpF-atpD:* 4nt  *psbC-psbD:* 53nt  *rpl4-rpl23:* 8nt | *sufC-sufB:* 1nt  *atpF-atpD:* 4nt  *psbC-psbD*: 53nt  *rpl4-rpl23:* 8nt | *sufC-sufB:*  1nt  *atpF-atpD*:  4nt  *psbC-psbD:* 53nt  *rpl4-rpl23:*  8nt | *sufC-sufB:*  1nt  *atpF-atpD:* 4nt  *psbC-psbD:* 53nt  *rpl4-rpl23:* 8nt | *sufC-sufB:* 1nt  *atpF-atpD:* 4nt  *psbC-psbD:* 53nt  *rpl4-rpl23:* 8nt | *sufC-sufB:*  1nt  *atpF-atpD:*  1nt  *psbC-psbD:* 53nt  *rpl4-rpl23:*  8nt |

Abbreviation: *Thalassiosira (T.), Cyclotella (Cy.), Roundia (Ro.), Chaetoceros (Ch.), Cerataulina(Ce.), Rhizosolenia(Rh.)*

a: missing *petF*, has orf127

b: *ycf66* is a pseudogene

c: missing *ycf42*, has *acpP1* and *syfB*

d: missing *ycf42*, has *acpP1* and *syfB*, *ilvB*, *ilvH*

e: missing *psaE*, *psaI*, *psaM*, *ycf35*, *tufA*, *syfB*, has *acpP1*.
