# Supplementary material for: Conserved Gene Order and Expanded Inverted Repeats Characterize Plastid Genomes of Thalassiosirales
Source: PLoS One. 2014 Sep 18;9(9):e107854. doi: 10.1371/journal.pone.0107854 (PMC4169464; doi:10.1371/journal.pone.0107854)
Supplement: Table S5 — Predicted repeat pairs in seven sequenced diatom plastid genomes. (DOCX) [file pone.0107854.s008.docx]

**Table S5.** Predicted repeat pairs in seven sequenced diatom plastid genomes.

| Species | Identity | Alignment length | Number of mismatches | Number of gap opens | Start1 | End1 | Start2 | End2 | E-value | Bit score |
| --- | --- | --- | --- | --- | --- | --- | --- | --- | --- | --- |
| *Cy. sp. W03_2* | 100 | 84 | 0 | 0 | 65293 | 65376 | 65211 | 65294 | 1e^-36^ | 152 |
| *Cy. sp. W03_2* | 100 | 82 | 0 | 0 | 83554 | 83635 | 83472 | 83553 | 2e^-35^ | 149 |
| *Cy. sp. L04_2* | 100 | 79 | 0 | 0 | 65268 | 65346 | 65190 | 65268 | 7e^-34^ | 143 |
| *T. oceanica* | 96.67 | 90 | 3 | 0 | 29941 | 30030 | 18564 | 18475 | 2e^-35^ | 149 |
| *T. oceanica* | 91.25 | 80 | 7 | 0 | 6626 | 6705 | 5376 | 5297 | 1e^-24^ | 113 |

Generic abbreviations are: *Cyclotella* (*Cy.*), *Thalassiosira* (*T.*).
