# Supplementary material for: Conserved Gene Order and Expanded Inverted Repeats Characterize Plastid Genomes of Thalassiosirales
Source: PLoS One. 2014 Sep 18;9(9):e107854. doi: 10.1371/journal.pone.0107854 (PMC4169464; doi:10.1371/journal.pone.0107854)
Supplement: Table S6 — The permutation of number coded Locally Colinear Block (LCB) for each plastid genome. Negative number indicates an inversion of the given LCB. (DOCX) [file pone.0107854.s009.docx]

**Table S6.** The permutation of number coded Locally Colinear Block (LCB) for each plastid genome. Negative number indicates an inversion of the given LCB.

| *Rhizosolenia imbricata* | -1 -2 -3 -4 -5 -6 -7 -8 -9 -10 -11 -12 13 14 15 16 -17 18 19 20 21 -22 -23 24 -25 -26 -27 -28 -29 30 31 32 |
| --- | --- |
| *Chaetoceros simplex* | -4 12 1 11 10 9 8 13 5 7 -3 2 -16 -15 -14 17 6 18 19 20 21 30 31 32 27 26 25 -24 23 -22 -28 -29 |
| *Cerataulina daemon* | 1 -17 12 11 10 9 8 13 5 7 3 2 -16 -15 -14 -4 6 18 19 20 21 29 28 22 30 31 32 27 26 25 -24 23 |
| *Cyclotella nana* | 1 10 9 14 15 19 20 8 12 11 6 18 17 16 13 5 7 4 3 2 21 29 28 22 23 24 30 31 32 27 26 25 |
| *Thalassiosira weissflogii* | 1 10 9 14 15 19 20 8 12 11 6 18 17 16 13 5 7 4 3 2 21 29 28 22 23 24 30 31 32 27 26 25 |
| *Roundia cardiophora* | 1 10 9 14 15 19 20 8 12 11 6 18 17 16 13 5 7 4 3 2 21 29 28 22 23 24 30 31 32 27 26 25 |
| *Cyclotella sp.W03_2* | 1 -19 -15 -14 -9 -10 20 8 12 11 6 18 17 16 13 5 7 4 3 2 21 29 28 22 23 24 30 31 32 27 26 25 |
| *Cyclotella sp. L04_2* | 1 -19 -15 -14 -9 -10 20 8 12 11 6 18 17 16 13 5 7 4 3 2 21 29 28 22 23 24 30 31 32 27 26 25 |
| *Thalassiosira oceanica* | 1 10 -15 -14 -21 -20 -16 9 18 17 -7 -5 4 3 -11 -12 -13 -8 6 19 2 22 31 30 -25 -24 -23 -29 27 -32 -26 33 28 |

Note: Only one IR is included in this analysis.

Highlighted area indicates the one single inversion between *Roundia cardiophora* plastid genome and *Cyclotella* sp. W03_2 and *Cyclotella* sp. L04_2 plastid genomes.
