# Supplementary material for: Conserved Gene Order and Expanded Inverted Repeats Characterize Plastid Genomes of Thalassiosirales
Source: PLoS One. 2014 Sep 18;9(9):e107854. doi: 10.1371/journal.pone.0107854 (PMC4169464; doi:10.1371/journal.pone.0107854)
Supplement: Table S7 — Pairwise number of inversions inferred by GRIMM. (DOCX) [file pone.0107854.s010.docx]

**Table S7.** Pairwise number of inversions inferred by GRIMM (Tesler, 2002)

|  | *T.*  *weissflogii* | *Cy. sp. L04_2* | *Cy. sp.*  *WC03_2* | *Cy.*  *nana* | *T. oceanica* | *Ro. cardiophora* | *Ch. simplex* | *Ce. daemon* | *Rh. imbricata* |
| --- | --- | --- | --- | --- | --- | --- | --- | --- | --- |

| ***T. weissflogii*** |  |  |  |  |  |  |  |  |  |
| --- | --- | --- | --- | --- | --- | --- | --- | --- | --- |
| ***Cy. sp. L04_2*** | 1 |  |  |  |  |  |  |  |  |
| ***Cy. sp.WC03_2*** | 1 | 0 |  |  |  |  |  |  |  |
| ***Cy. nana*** | 0 | 1 | 1 |  |  |  |  |  |  |
| ***T. oceanica*** | 10 | 11 | 11 | 10 |  |  |  |  |  |
| ***Ro. cardiophora*** | 0 | 1 | 1 | 0 | 10 |  |  |  |  |
| ***Ch. simplex*** | 17 | 18 | 18 | 17 | 22 | 17 |  |  |  |
| ***Ce. daemon*** | 14 | 15 | 15 | 14 | 19 | 14 | 8 |  |  |
| ***Rh. imbricata*** | 20 | 21 | 21 | 20 | 25 | 20 | 14 | 12 |  |

Abbreviation: *Thalassiosira (T.), Cyclotella (Cy.), Roundia (Ro.), Chaetoceros (Ch.), Cerataulina(Ce.), Rhizosolenia(Rh.).* The zero inversion in yellow indicates those three plastid genome *Cy. nana*, *T. weissflogii* and *Ro. cardiophora* have the same gene order.
