# Supplementary material for: Conserved Gene Order and Expanded Inverted Repeats Characterize Plastid Genomes of Thalassiosirales
Source: PLoS One. 2014 Sep 18;9(9):e107854. doi: 10.1371/journal.pone.0107854 (PMC4169464; doi:10.1371/journal.pone.0107854)
Supplement: Table S8 — Genes at the boundary of each Locally Colinear Block (LCB). (DOCX) [file pone.0107854.s011.docx]

**Table S8.** Genes at the boundary of each Locally Colinear Block (LCB)

| **LCB number** | **Genes names** |
| --- | --- |
| 1 | psaA, psaB |
| 2 | psaF, psaJ |
| 3 | ycf90, psbI |
| 4 | petB, psaD |
| 5 | psbD, psbC |
| 6 | secG, psaM |
| 7 | ycf12, psbZ |
| 8 | dnaB, rpl12 |
| 9 | psbX, psbV |
| 10 | rpl19, ssra |
| 11 | petA, ycf3  *rps1* |
| 12 | rps18, rps2 |
| 13 | psbK, psaI |
| 14 | rbcS, atpA |
| 15 | psbB, psbH |
| 16 | petN, ycf33 |
| 17 | petG |
| 18 | rps14, ftsH |
| 19 | psaE, rpl20 |
| 20 | ycf45, acpP |
| 21 | ycf89, rrn5 |
| 22 | psbA |
| 23 | psaC |
| 24 | ccsA |
| 25 | rps6, thiG |
| 26 | clpC |
| 27 | rps10, rps12 |
| 28 | ccs1, ycf46 |
| 29 | rpl34, rpl32 |
| 30 | rps16, groEL |
| 31 | dnaK, rpl16 |
| 32 | rpl18, rpl31 |
